# *Hyalomma anatolicum* as the Main Infesting Tick in an Important Livestock Rearing Region, Central Area of Iran

**Published:** 2018-05

**Authors:** Peyvand BIGLARI, Hasan BAKHSHI, Sadegh CHINIKAR, Hamid BELQEISZADEH, Masoud GHAFFARI, Siavash JAVAHERIZADEH, Faezeh FAGHIHI, Zakkyeh TELMADARRAIY

**Affiliations:** 1. Dept. of Biology Biosystematics, Faculty of Modern Medical Sciences, Islamic Azad University, Tehran Medical Sciences Branch, Tehran, Iran; 2. Malaria and Vector Research Group, Biotechnology Research Center, Pasteur Institute of Iran, Tehran, Iran; 3. Laboratory of Arboviruses and Viral Hemorrhagic Fevers (National Reference Laboratory), Pasteur Institute of Iran, Tehran, Iran; 4. Dept. of Parasitology and Entomology, Islamic Azad University, Tehran Medical Sciences Branch, Tehran, Iran; 5. Veterinary Office of Golpayegan, Isfahan, Iran; 6. Dept. of Clinical Laboratory Sciences, Faculty of Paramedical Sciences, Islamic Azad University, Tehran Medical Sciences Branch, Tehran, Iran; 7. Cellular and Molecular Research Center, Iran University of Medical Sciences, Tehran, Iran; 8. Dept. of Medical Entomology and Vector Control, School of Public Health, Tehran University of Medical Sciences, Tehran, Iran

**Keywords:** Ixodidae, *Hyalomma anatolicum*, Livestock, Iran

## Abstract

**Background:**

This study was carried out to determine the infestation of domestic ruminants to ticks in an important livestock-rearing region, located in central part of Iran.

**Methods:**

Ticks were collected from cattle, sheep, and goats and then were identified with appropriate identification keys to species level in two different ecological regions of plains and mountain in 4 seasons in 2015.

**Results:**

Totally 492 ticks from cattle, sheep, and goats in 34 herds were collected. Totally, 18.53% of domestic animals were infected by ticks. All ticks were belonged to family Ixodidae and classified into three genera and six species comprising *Hyalomma anatolicum* (38.83%)*, Hy. Asiaticum* (23.37%), *Hy. marginatum* (2.85%), *Hy. sp.* (3.45%), *Rhipicephalus sanguineus* (14.02%) and *Haemaphysalis sulcata* (10.98%). Sex ratio of the collected specimens showed 241 (48.99%) male, 219 (44.51%) female and 32 (6.5%) nymph

**Conclusion:**

Studied area is important for production of livestock and dairy products. Annually, many livestock products are exported to other parts from this region; therefore, it is very important to identify the infection rate of tick-borne diseases as well as safety factors on livestock.

## Introduction

Ticks are ectoparasites, living by hematophagy on the blood of birds, mammals, reptiles, and amphibians. Some of tick species act as vectors of a broad range of pathogens of domestic animals like sheep and goats and are responsible for damage directly due to their feeding behavior (1). Ticks can transmit a variety of diseases such as Crimean Congo hemorrhagic fever (CCHF), anaplasmosis, babesiosis, rickettsiosis, borreliosis and ehrlichiosis in which CCHF is considered as one of the most deadly arboviruses (2).

Tick studies in Iran were initiated by Delpy and then Later, Abbasian-Lintzen and Mazlum compiled a list of ticks collected from domestic animals. In another investigation, data for ixodid ticks were taken from mammals, mainly rodents in different locations of the country. Ticks parasitizing wild sheep and goats were studied in the country and currently, prevalence of ticks was studied in the northwest and the western part of Iran (3, 4).

Distribution of various species of ticks on domestic animals in some geographical locations of Iran was studied (5). Due to the importance of ticks and tick-borne diseases, there are many reports on epidemiology, distribution and medical importance of different ticks through the country (6, 7).

Due to geographical location, climate, topography and diversity, Golpayegan County is a major hub of livestock rearing in Iran. This county is located in Isfahan Province, central part of the country. The current study aimed to investigate bio-systematically the distribution of ticks based on their genus and species. Due to the importance of animals’ husbandry and dairy products, understanding the distribution of ticks provides important data for preventing tick-associated diseases in livestock. Study on distribution of ticks, which infect the domestic ruminants provide a clue for tick-borne diseases in the region (8).

This study was conducted to determine the tick infestation status in domestic ruminants in Golpayegan County, Isfahan Province, central part of Iran in 2015.

## Methods

### Study area

**Fig. 1:**
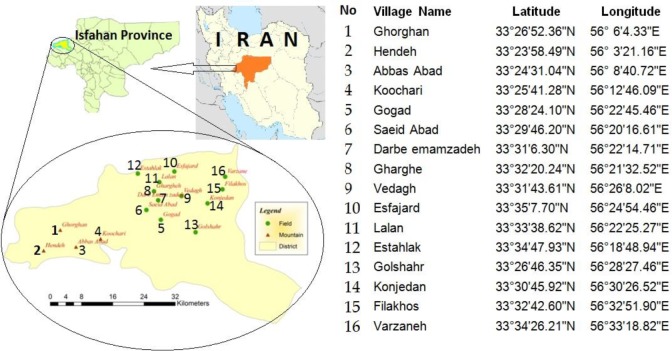
Details of sampling regions and the latitudes and longitudes of the studied places

Isfahan Province covers an area of approximately 107000 km^2^. Golpayegan County (33°27’, 50°18’ E) is located in this province (Fig. 1). The mean elevation of this city is 1800m above sea level and the average annual rainfall is 300 mm. According to the census of the veterinary office in 2014, Golpayegan County has about 25000 cattle, 105000 sheep, and 15000 goats.

### Tick collection

From July 2014 to June 2015, 16 villages in two different ecological regions including 12 plains (70%) and 4 mountain (30%) regions were randomly selected as the study area (Fig. 1).

We used the formula n0=Z1−a22p(1−p)d2 ((1−p)=0.7, z1−a2=1.96) for calculation of the sample size (9, 10). In total, 437 livestock including 208 sheep, 55 goats, and 174 cattle were selected randomly and examined individually for tick infestation. Thirty minutes were spent for each flock to collect ticks. The entire body of each animal including ears, nape of the neck, perineum, scrotum, and the tail base was inspected for the presence of tick species. Collected ticks were kept alive in labeled vials and then transferred to the laboratory in School of Public Health, Tehran University of Medical Sciences (TUMS), Tehran, Iran for species identification by using the appropriate identification keys (11, 12).

## Results

During the study period among 16 villages in two different ecological regions, 492 ticks including 460 adults and 32 nymphs (some ticks included eggs) were collected. For detection of infestation, 208 sheep, 174 cattle and 55 goats in 34 herds were examined for presence of ticks. Out of all livestock, 18.53% (81 out of 437) were infested with a total number of 492 ixodid ticks (Table 1). The tick species diversity in the cattle (49.39%) was significantly higher than two other hosts whereas goats (13.58%) had the least tick species diversity. The mean number of ticks on each animal was 6.07 ticks per animal.

**Table 1:** The frequency of examined ruminants and their infectivity rates to ticks in Golpayegan County, Isfahan Province, Iran

***Host Examined***	***No. (%)***	***Infestation per livestock (%)***	***Isolated ticks (%)***
Sheep	208 (47.60)	30 (14.4)	151 (37.03)
Cattle	174 (39.81)	40 (23)	301 (49.39)
Goats	55 (12.59)	11 (20)	40 (13.58)
Total	437 (100)	81 (18.53)	492 (100)

Totally, 3 genus and 6tick species were identified comprising: *Hyalomma anatolicum* (38.83%)*, Hy. asiaticum* (23.37%), *Hy. Marginatum* (2.85%), *Hyalomma sp.* (3.45%), *Rhipicephalus sanguineus* (14.02%) and *Haemaphysalis sulcata* (10.98%). Out of all collected ticks, 369 (75%) of tick samples belonged to *Hyalomma* genus. *Hyalomma anatolicum* (38.83%) was the most abundant species while *Hy. marginatum* (2.85%) was the least species. Sex ratio of the collected specimens showed 241 (48.99%) male, 219 (44.51%) female and 32 (6.5%) nymph (Table 2).

**Table 2:** The distribution of relative frequency of collected ticks according to species from animals divided to different sexes in Golpayegan County, Isfahan Province, Iran

***Species***	***Male/Female***			
	**Male**		**Female**	
	**No.**	**%**	**No.**	**%**
*Hyalomma anatolicum*	125	51.87	66	30.14
*Hyalomma asiaticum*	60	24.9	55	25.11
*Hyalomma marginatum*	8	3.31	6	2.74
*Hyalomma* sp.	-	-	17	7.76
*Rhipcephalus sanguineus*	26	10.79	43	19.64
*Haemaphysalis sulcata*	22	9.13	32	14.61
Total (Only Adults)	241 (48.99)	100	219 (44.51)	100
Nymph	32(6.5)			
Total (Adults+Nymphs)	492 (100)			

The abundance of isolated tick species from infested ruminants related to topographical zones of Golpayegan district is summarized in Table 3. Overall, 447 (90.85%) tick species collected from plateau zone and the rest were from mountain zone. The species diversity of hard ticks in plateau areas was more than mountain area. *Hyalomma anatolicum* and *Rhipicephalus sanguineus* species were the dominant species of ticks in plain and mountain zones respectively. *Hy. anatolicum*, *R. sanguineus* and *Hyalomma nymph* occurred in both plain and mountain areas. *Hy*. *asiaticum*, *Hy*. *marginatum, Hyalomma* sp. and *H. sulcata* occurred in plateau areas only. *Hyalomma anatolicum* (191:38.83%) was the most frequent species in both plain and mountain areas whereas *Hy. marginatum* was the least species.

**Table 3:** The abundance of isolated tick species from infested ruminants related to topographical zones types in Golpayegan County, Isfahan Province, Iran 2014–2015

***Tick species***	***Plateau***		***Mountain***		***Frequency***	
	**No.**	**%**	**No.**	**%**	**No.**	**%**
*Hyalomma anatolicum*	184	37.4	7	1.43	191	38.83
*Hyalomma asiaticum*	115	23.38	–	–	115	23.37
*Hyalomma marginatum*	14	2.85	–	–	14	2.85
*Hyalomma* sp.	17	3.45	–	–	17	3.45
*Hyalomma* nymph	31	6.3	1	0.2	32	6.50
*Rhipicephalus sanguineus*	32	6.5	37	7.52	69	14.02
*Haemaphysalis sulcata*	54	10.97	–	–	54	10.98
Total	447	90.85	45	9.15	492	100

The frequency result of collected ticks in four seasons revealed that more about half (51.82%) of specimens have been collected in spring, 23.18% in summer, 14.03% in autumn and 10.97% in winter. Prevalence of isolated ticks species from infested ruminants related to different seasons in the study area is summarized in Table 4.

**Table 4:** Prevalence of isolated tick species from infested ruminants related to different seasons in Golpayegan County, Isfahan Province, Iran

***Tick species***	***Plateau***				***Mountain***				***Total***
	**Spring**	**Summer**	**Autumn**	**Winter**	**Spring**	**Summer**	**Autumn**	**Winter**	
*Hy. anatolicum*	86	47	51	–	6	1	–	–	191
*Hy. asiaticum*	89	14	12	–	–	–	–	–	115
*Hy. marginatum*	8	2	4	–	–	–	–	–	14
*Hy.* sp.	4	13	–	–	–	–	–	–	17
*Hy Nymph*	–	29	2	–	–	1	–	–	32
*R. sanguineus*	27	5	–	–	35	2	–	–	69
*H. sulcata*	–	–	–	54	–	–	–	–	54
Total	214	110	69	54	41	4	–	–	492

## Discussion

Ticks are subject to many studies in Iran, but there is limited tick-related information in some geographical areas including central part of the country (13). In the current study, 492 ticks were collected from domesticated hosts including cattle, sheep, and goats in 34 herds in both plain and mountain areas of an important livestock-rearing region in central part of Iran. Totally, 18.53% of livestock (81 out of 437) were infested with a total number of 492 ixodid ticks. The most and least infested cases were reported from plateau and mountain zones, respectively. In Iran, this rate was 11.14% in Ilam Province, west of the country in which has a borderline with Iraq (14), 9.37% in Hamedan Province, west of Iran (6), 24% in Mazandaran Province, north of the country (10), 43% in Golestan Province, north of Iran (15), 16.3% in Tehran Province, capital of Iran (16), 28.59% in Darreh Shahr in Ilam Province (17) and 27% in Kashan located in central part of Iran (18, 19). The infection rates based on the animal host is consistent with the results of Golestan (15) and Tehran (16), but it is contrary to results on different hosts in different geographical areas. The controversy in the results might be associated to different parameters including the quality of animals’ nourishment, the variance in the cattle’s sensitivity to different ticks’ strains, cattle’s hygiene and applying preventive strategies including cattle’s spraying and anti-ticks bath. The mean number of ticks on each animal was low (about 6.07 ticks per animal). The occurrence of ticks on cattle, sheep, and goats were 61.18%, 30.7% and 8.12% respectively. Through viewpoint of study were conducted about tick infestation of livestock, the percentage of tick per animal in different geographical areas of Iran such as Golestan (15), Mazandaran (10), Darreh Shahr (17), Isfahan (18, 19), Kermanshah (20) and (5), infestation rate on cattle with studies in Ilam (17) and in four different zoogeographical areas in Iran (5) was coincided but in other parts of Iran the highest infestation rate related to sheep host. Three genera were collected in this investigation where the *Hyalomma* species comprised 75% of our collected samples comprising *Hy. anatolicum, Hy. marginatum* and *Hy. asiaticum*. These results are in agreement with the study conducted in West Azerbaijan, Zabol, Ghaen, Abdanan, Esfahan, Ardebil, Sarepole-zahab, Ghom and Meshkin-Shahr districts (3, 6, 9, 19, 21–26). *Hyalomma* ticks were dominant in the investigated area. In another study directed in Golestan and Ghazvin, *Rhipicephalus* (10, 15, 27) and in Guilan, *Boophilus* (28) were the most abundant ticks respectively. In our survey, *Hyalomma anatolicum* (38.83%) was the most abundant species while *Hy. marginatum* (2.85%) was the least. In studied accomplishes in neighboring of Iran such as Turkey, Iraq, Oman, Saudi Arabia, Kuwait, Yemen, Qatar, United Arabic Emirates and Bahrain, the most abundant collected ticks from livestock was genus *Hyalomma* (29–34). Based on our data and comprising them with the same investigations in the field, *Hyalomma* species is the dominant tick species in the Middle East.

In the current study, the most diversity has been observed among the genus *Hyalomma* by 3 species by 75% prevalence. Between collected ticks, *Hyalomma* ticks have the most number and frequency of species and display on each three different hosts (cattle, sheep and goats). Frequency of tick specimens during different seasons was different. The frequency of the species of genus *Hyalomma* was higher than the others and *Hy. anatolicum* was the most frequent species. The result of the current study is in agreement with studies of Bushehr located in south of the country (35), Mash-had, east of Iran (36), Isfahan (19), Mahabad, north west of the country (37), Kurdistan, west of Iran (38), West-Azerbaijan (39), the study conducted in Iran (40) and in north and south of the country (41) and studied accomplishes in neighboring of Iran such as Iraq, Pakistan and India (31, 32, 42). The results of frequency of collected ticks in four seasons state that more than half (51.82%) of specimens have been collected in spring, 23.18% in summer, 14.03% in autumn and 10.97% in winter. We observed that infestation rate maximized in spring and summer. Most of the ticks were isolated from early in Apr to middle of Sep, when the mean temperature and humidity have been between 21.5 and 32.5 ^°^Cand 43% to 56%, respectively. During these months, there is suitable vegetation in pasture for grazing livestock and the climatic condition is favorable for complete life cycle of tick. Prevalence of isolated ticks species from infested ruminants was the most in spring season (51.82%) and the least in winter season (10.97%) which coincided with results in Urmia, north-west of Iran (43), Oshnavieh suburb, west of Iran (44), Yazd, central part of the country (18), Ilam (14) and Hamedan (4). Currently, in an investigation conducted in northwestern Iran, the most of ticks were collected in summer and spring respectively (39).

In the present study, most of tick species were collected from cattle (dominant species: *Hy. anatolicum*) in spring and *Ha. sulcata* were found on sheep in winter. The results of prevalence of isolated ticks from infested ruminants related to different seasons reveal that the most number of tick species are in plateau zone and spring season.

## Conclusion

Studied area is considered as one of the most important centers of agriculture and livestock rearing in the region. This district is also important for dairy products. Because of region’s landscape, weather, and livestock, the dairy products have earned a high rank in the county. Annually, many livestock products are exported to other parts of Iran; finally, the identification of the infection rate of tick-borne diseases as well as safety factors on livestock is suggested.

## Ethical considerations

Ethical issues (Including plagiarism, informed consent, misconduct, data fabrication and/or falsification, double publication and/or submission, redundancy, etc.) have been completely observed by the authors.
